# Work–Family Interface Profiles and Their Associations with Personal and Social Factors among South Korean Dual-Earner Parents

**DOI:** 10.3390/bs14100887

**Published:** 2024-10-01

**Authors:** Yangmi Lim

**Affiliations:** Department of Home Economics Education, College of Education, Jeonju University, Jeonju 55069, Republic of Korea; ym68@jj.ac.kr; Tel.: +82-63-220-2338

**Keywords:** work–family interface profiles, conflict, enrichment, dual-earner parents, latent profile analysis, personal and social factors

## Abstract

The work–family interface literature has focused on a variable-centered approach, and few studies have used a person-centered approach to investigate work–family interface types and their associations with psychosocial factors. This study explored whether distinct work–family interface types could be identified at a dyadic level in dual-earner couples by combining work–family conflict (WFC) and enrichment (WFE) for both parents. It also examined how these couples’ comprehensive types of work–family interface were related to psychosocial outcomes. Conducting a latent profile analysis in a sample of 558 dual-earner couples (*M_age_*: 40.43 ± 4.07 years for fathers, 37.97 ± 3.57 years for mothers) with first-grade children in elementary schools participating in the Panel Study on Korean Children, this study identified three work–family interface profiles: *Beneficial fathers/Moderate active mothers* (fathers reporting low WFC and high WFE/mothers reporting moderate WFC and WFE), *Beneficial* (both parents reporting low conflict and high enrichment), and *Harmful* (both parents reporting high conflict and low enrichment). Fathers’ education, household income, and social support influenced their membership in work–family interface profiles. Overall, members with *Beneficial fathers/Moderate active mothers* and *Beneficial* profiles showed more positive personal and family outcomes than those with *Harmful* profiles.

## 1. Introduction

Family, work, and health are the most important aspects of adulthood. Family and work were considered separate spheres in the early and mid-20th centuries, but they have gradually been recognized as influencing each other in mutual and bidirectional ways [1]. With this recognition, the term “work–family interface” emerged, which refers to the experiences that arise from simultaneously performing the roles required in work and family life [2].

Research has investigated the work–family interface over the past decades owing to its relevance to organizational and individual functioning. Despite the proliferation of research, some limitations remain in our understanding of work–family interface [3]. This study addresses some of these limitations. First, the work–family interface literature has largely focused on negative ways in which work and family intersect; thus, more studies that conceptualize the work–family interface as involving the co-occurrence of negative and positive aspects are needed. Second, prior research in this domain has been primarily based on a variable-centered approach—the traditional and dominant approach in social sciences. Its purpose is to explain the relationships between variables of interest across populations. However, this approach may overlook nuanced differences between individual participants or groups within the data. By contrast, a person-centered approach focuses on how a set of chosen variables combines within individuals, extracting distinct subpopulations (i.e., profiles or classes) from these variables [4]. The latter approach is advantageous for seeking tailored interventions because it is possible to identify differences in the groups produced by combining specific variables and other variables that affect group membership [5]. Third, work–family interface research has typically focused on individual-level analyses. Although empirical studies show that work–family role sharing is a major task that dual-earner couples should accomplish [6], few attempts have been made to adopt a dyadic approach (except for Vieira et al.’s study [3]).

The current study aimed to fill these gaps by exploring whether work–family interface types, acquired from specific combinations of positive and negative work–family interactions in South Korean dual-earner couples, can be identified at a dyadic level. This study also examined which social and economic factors influence the membership of work–family interface types and how these couple-level types are related to parent- and child-related outcomes.

### 1.1. Different Approaches to Measuring Work–Family Interface

There are a wide variety of views regarding the measurement of work–family interface [7]. These views can be divided into two categories. Originally, scholars introduced the “overall appraisal approach” to measure individuals’ subjective evaluation on work–family interface by asking a general single-item question about how successful one feels in balancing work and family life. Next, the “components approach” followed, which posited that work–family interface comprises two components, work–family conflict (WFC) and work–family enrichment (WFE) [8]. WFC and WFE represent the negative and positive aspects of the work–family relationship, respectively.

The scarcity hypothesis [9] and role stress theory [10] serve as theoretical bases for WFC. The scarcity hypothesis suggests that individuals have a finite amount of time, energy, and attention. Therefore, the more roles an individual occupies, the more likely it is that those limited resources will become depleted [9]. Additionally, according to the role stress theory proposed by Kahn et al. [10], role conflict is defined as the simultaneous occurrence of two (or more) sets of role pressures such that compliance with one would make compliance with the other more difficult. Drawing on the scarcity hypothesis [9] and role stress theory [10], WFC is defined as a form of inter-role conflict or perceived stress that occurs from fulfilling the simultaneous and conflicting demands required at work and home owing to limited resources such as energy and time. WFC is found to be caused by factors such as overtime, job instability, and the burden of childcare [11]. However, the concept of WFE can be derived from the role accumulation theory [12] and the enhancement hypothesis [13]. According to role accumulation theory, participation in multiple roles brings a series of benefits to individuals [12]. Similarly, the enhancement hypothesis proposes that participation in one role brings about rewards and privileges that enhance performance in other roles [13]. Therefore, WFE refers to the extent to which resources (e.g., income and social skills) gained from participation in one role domain (e.g., work) enhance positive experiences in the other role domain (e.g., family) [14]. WFE is facilitated by social support factors such as family-friendly organizational policies (e.g., extended parental leave and increased working time flexibility) [11]. Thus, work–family balance can be perceived as low WFC and high WFE [15].

### 1.2. Typology of Work–Family Interface

Previous components approach–based research has primarily investigated WFC and WFE separately. However, the components approach dismissed the fact that an individual can simultaneously experience different combinations of WFC and WFE (i.e., both are low; WFC is low—WFE is high) [16]. To address this limitation, a few pioneering studies [17,18] have identified the typology of the work–family interface by adopting a person-centered approach. Four different types of work–family interfaces have emerged as part of this typology, characterized by different WFE and WFC, and labeled in various ways, which is illustrated in Table 1. First, the types of work–family interfaces characterized by (1) high WFC and low WFE or (2) high WFE and low WFC have been named *Imbalanced* vs. *Balanced* [17] or *Harmful* vs. *Beneficial* [18], respectively. As suggested by these names, the type of high WFC and low WFE has a low level of work–family balance and overall harmful effects on an individual. Conversely, the type of low WFC and high WFE has a high level of work–family balance and beneficial effects on an individual. In addition, low engagement across work and family roles (low WFC and low WFE) has been labeled *Segmented* [17] or *Passive* [18]. This type was assumed by Rantanen et al. [18] to have low engagement and indifference across work and family roles because the obligations attached to work and family domains are not particularly rewarding. In such a situation, individuals tend to be passive in performing their work and family roles. In addition, Grzywacz et al. [17] argue that workers belonging to this type consider work and family to be completely separate (or segmented) domains with no influence on each other because of the impermeable boundaries between them. However, high engagement across work and family roles leads to *Blurred* [17] or *Active* [18] types. According to Grzywacz et al. [17], workers belonging to this type consider the boundaries between the work and family domains permeable and experience work–family role blurring, referred to as a situation under which the work and family roles are highly integrated, causing individuals to struggle in distinguishing between them. Rantanen et al. [18] claim that workers of this type show active engagement and attentiveness in both work and family roles.

More recently, studies have been conducted with various employees and cultures using advanced statistical methods (e.g., latent profile analysis [LPA]). However, these studies have classified the types of work–family interface by closely comparing statistical values (e.g., means) of WFE and WFC among the identified types according to their respective criteria and produced inconsistent findings [19,20,21]. In a study of Portuguese bankers, Carvalho and Chambel [19] found five profiles, three of which (*Beneficial* [46.5%], *Harmful* [2.3%], and *Passive* [3.9%]) were consistent with earlier research [17,18]. However, two distinct profiles were discovered: *Moderate active* (9.4%, with moderate levels of WFC and WFE) and *Moderate harmful* (37.8%, with moderate WFC and low WFE). The *Beneficial* and *Moderate harmful* profiles were the most prevalent. Robinson et al. [20] showed five types of work–family interfaces among Australian working mothers: *Active* (33.7%), *Negative active* (23.5%, with lower scores on WFE compared to the *Active* type), *Beneficial* (22.1%), *Fulfilled* (11.7%, with higher scores on some items on WFE than the *Beneficial* type), and *Harmful* (9.1%). Of the five types, the *Active*, *Negative active*, and *Beneficial* types were the most common. However, in a sample of Chinese university counselors, Zhang et al. [21] found three profiles of work–family interfaces: *Slightly conflictual* (51%, with slightly higher than average WFC, slightly lower than average WFE), *Enriched* (34%, with high WFE, low WFC), and *Work-to-family conflictual* (15%, with high work-to-family conflict, low family-to-work conflict, and WFE).

Unlike previous studies that identified the work–family interface types of individual workers, Vieira et al. [3] reported four different couple-level profiles in the work-to-family and family-to-work balance directions among Portugal’s dual-earner parents with preschool children. Specifically, in the work-to-family balance direction, *Harmful* (20%), *Beneficial* (29.3%), *Active* (29.5%), and *Harmful men/Beneficial women* (21.1%, a dissimilar interface pattern for husbands and wives) groups were identified; and in the family-to-work balance direction, *Harmful* (33.7%), *Beneficial* (13.9%), *Passive* (36.2%), and *Beneficial men/Harmful women* (16.2%, a dissimilar interface pattern for husbands and wives) groups were also found.

### 1.3. Social and Personal Factors Related to Work–Family Interface Types

Prior research has also explored personal and social factors related to work–family interface types, which can be organized into three aspects. First, several scholars [20,21] argued that the typology of the work–family interface appears differently depending on the gender roles and culture of employees. Women experience different combinations of WFC and WFE compared to men. Despite the fact that dual-earner families are a common family pattern in many societies, traditional gender-role-based expectations are still prevalent, with women continuing to be mainly responsible for childcare and housework and men’s paid work still being more valued than women’s work [3]. Therefore, in dual-income families, women are more likely than men to experience WFC [6].

Moreover, in some countries in East Asia, such as South Korea and China, the *Harmful* type may be the most prevalent owing to longer working hours and demanding work duties [21]. For instance, among the countries in the Organisation for Economic Co-operation and Development (OECD), South Korea has one of the highest average annual working hours [22], whereas South Korea’s work–life balance index ranks among the lowest of the OECD countries [23]. In a survey conducted in South Korea, most South Korean workers (men: 69.6%; women: 67.0%) experienced WFC, with most causes being work-related, such as overtime work [11]. Additionally, unlike Western contexts, in which work has often been regarded as a separate and even conflicting area with family, in Confucian collectivistic cultures, prevalent in China and South Korea, work is a critical tool that enhances the financial welfare and social status of the family, which can lead to a blurring of the boundaries between work and family. Individuals in Confucian collectivistic cultures tend to perceive the economic gains acquired from increased workloads as contributing to family wealth. Therefore, they feel less stressed about the loss of personal time spent with their families [24,25]. Thus, the *Active* type may also be dominant in certain East Asian countries (e.g., South Korea). Comprehensively, despite the possibility that gender roles and culture may influence the typology of the work–family interface, there is a dearth of empirical research verifying this.

Second, some research has identified the social and economic factors influencing the membership of specific work–family interface types; however, the findings remain incompatible. Jung et al. [26] revealed three work–family interface profiles among dual-income South Korean parents: *Beneficial* (both parents, 26.6%), *Harmful* (both parents, 40.3%), and *Beneficial fathers/Harmful mothers* (33.1%). They also found that dual-earner parents whose fathers had full-time jobs were more likely to be in the *Harmful* than *Beneficial fathers/Harmful mothers* profile. However, Sung and Ki [27], who also investigated the typology of the work–family interface among South Korean working mothers, reported that the higher the mothers’ education level, the higher the likelihood of being members of the *Beneficial* type. Social support can also provide individuals with a variety of resources such as positive emotions, more time, and new knowledge or skills—all of which can reduce WFC and increase WFE [28]. The more help dual-earner parents receive from others in raising their children, the more likely they are to belong to the *Beneficial* rather than *Harmful* type [26]. In addition, the perception of job control, turnover intentions, number of children, and household income also influenced the membership of work–family interface profiles [29,30].

Third, many studies have identified how work–family interface influences personal and relational outcomes. Specifically, WFC was associated with adverse psychosocial outcomes, such as lower job productivity, poorer health perception, higher marital conflict, negative parenting, and children’s behavioral problems, whereas WFE was liked to positive outcomes, such as higher job satisfaction, improved health, higher parental warmth, and lower marital conflict [31,32,33,34]. The positive and negative effects of work–family dynamics can be explained by the spillover and crossover processes posited by family systems theory. A spillover process refers to the drift of positive and negative emotions, values, and experiences within individuals from one area of life (e.g., work) to another (e.g., family). The crossover process involves the dyadic transmission of positive and negative experiences, emotions, and dispositions between intimately connected individuals who share the same social environment. The spillover and crossover processes occur in the same valence. For example, demands (or resources) in the work domain generate stress (or commitment), which potentially leads to negative (or positive) spillover effects on individual life in the family domain. WFC (WFE) has a negative (positive) crossover effect on a partner’s well-being. The crossover process occurs either directly through empathy or emotional contagion or indirectly through interaction in a relationship between two people who form emotional bonds and spend much time together [35].

Prior studies, which took a person-centered approach, reported that the groups with higher levels of WFE and lower levels of WFC showed more positive psychosocial outcomes than those with lower levels of WFE and higher levels of WFC. Specifically, Yucel et al. [7] reported that the *Beneficial* (high WFE and low WFC) and *Moderate beneficial* (moderate WFE and low WFC) groups showed higher job satisfaction than the *Moderate active* (moderate WFE and WFC) ones. Similarly, Rantanen et al. [16] found that the *Beneficial* type revealed higher subjective well-being than the *Contradictory* (high work-to-family conflict and low work-to-family enrichment in combination with low family-to-work conflict and high family-to-work enrichment) and *Active* (high WFE and WFC) types. However, the findings for the *Active* and *Passive* groups have not been consistent. Some studies have found that the *Active* group fell between the *Beneficial* and *Harmful* groups in terms of vigor at work, self-rated health, and life satisfaction [36]. Other studies found that both *Active* and *Beneficial* groups reported higher family and job satisfaction than the other identified groups [3]. Moreover, some studies concluded that the *Passive* group had lower well-being indicators, such as life satisfaction and physical health, than the *Beneficial* group [37], whereas others concluded that the *Passive* group did not differ in terms of quality of life from groups with high WFE [36]. According to Vieira et al.’s study [3], which comprehensively analyzed the work–family interface of couples, when husbands belong to the *Harmful* type and wives the *Beneficial* type, not only is the husbands’ satisfaction with work and family lower, but wives’ family satisfaction and satisfaction with their husbands are also adversely affected. This finding suggests that even if one partner does not show the *Harmful* pattern, he or she can still be negatively affected by the partner’s work–family interface style. This result aligns with the tenets of family systems theory and the crossover process by which an individual’s experience affects those of other family members [3].

### 1.4. Study Aims and Research Questions (RQs)

This study aims to identify dyadic-level work–family interface types by combining WFC and WFE experiences among South Korean dual-earner parents and their associations with personal and social factors that previous research suggested. As prior studies have identified the relationship between work–family interface types and individual and relational outcomes by mainly focusing on individual health, life, and family satisfaction [3,7,16], relatively few studies have examined the links between the typology of work–family interface and parenting and children’s psychosocial adjustment. However, according to the results of studies adopting a variable-centered approach, parents’ work–family interface influenced parenting behaviors and children’s psychosocial health [6,34]. In addition, the stress associated with being a dual-income parent in South Korea can increase when a child enters elementary school because of the growing demand for parental support to help children adjust to school and the lack of afterschool public care systems [6,38]. Therefore, this study focused on dual-income parents with children in the first grade of elementary school.

- RQ1. How are the work–family interface profiles of dual-earner parents classified?
- RQ2. Which social and economic factors (education, household income, number of children, employment status, and social support) influence the work–family interface profile membership?
- RQ3. Do parent- and child-related outcomes (parents’ perception of health, marital satisfaction, parental warmth/control, and children’s behavioral problems) differ by the work–family interface profiles of dual-earner parents?

## 2. Materials and Methods

### 2.1. Participants and Procedure

This study utilized data derived from the eighth wave of the Panel Study on Korean Children (PSKC), administered by the Korea Institute of Child Care and Education [39]. The PSKC began collecting data from parents and their newborn children by applying a stratified multistage sampling procedure in 2008; specifically, as the first sampling unit, nationwide medical institutions with over 500 deliveries were selected. Second, among 2562 parents who voluntarily agreed to participate in the study and their newborn children in selected medical institutions, data collection from 2078 households actually responding to the survey (response rate: 81.1%) began. Since then, the PSKC has conducted annual follow-ups with participants regarding the psychosocial characteristics of the parents, parenting-related practices, and the overall development of the children through mailed questionnaires and home visit interviews. The PSKC data collected between 2008 and 2021 are publicly available. Of the 1598 households that participated in the PSKC 8th wave (attrition rate: 23.1%), this study selected 558 triads of dual-earner parents and their first-grade children (281 boys and 277 girls; *M_age_* = 7.33 ± 0.13 years) in South Korean elementary schools who responded to all study variables as the final participants. The average age of the parents was 40.43 ± 4.07 years for fathers and 37.97 ± 3.57 years for mothers.

The socioeconomic characteristics of the study participants are described in Table 2. In particular, the average monthly household income was 5.26 million Korean Republic won (KRW; SD = 197.37, approximately equivalent to 3940.87 USD).

### 2.2. Measures

#### 2.2.1. Work–Family Interface

Work–family interface was measured with WFE and WFC using Marshall and Barnett’s [40] Work–Family Gains Scale (WFGS) and Work–Family Strains Scale (WFSS), which were translated into Korean by the PSKC research team. The WFGS comprises 11 items related to the benefits and positive feelings that arise from performing the roles required in work and family life (including parenting, e.g., “Having both work and family responsibilities makes me a more well-rounded person”). The WFSS comprises 15 items concerning inner conflict and stress experienced in reconciling work and family life (including parenting; “Because of the requirements of my job, my family time is less enjoyable and more pressured”). Each item is scored on a five-point scale ranging from *not at all true* (1) to *very true* (5). The possible score ranges are 11–55 points for the WFGS and 15–75 points for the WFSS, with higher scores of the WFGS indicating that parents experience greater benefits and positive feelings in reconciling work and family life. Higher scores on the WFSS indicate that parents experience higher stress in combining work and family life. The internal consistency of the scales as measured by Cronbach’s α was 0.91 and 0.90 for the WFGS of fathers and mothers, respectively; additionally, Cronbach’s α was 0.91 and 0.93 for the WFSS of fathers and mothers, respectively.

#### 2.2.2. Social Support

Social support was measured using a scale developed by the PSKC research team based on the scales used in the studies by Lee and Ok [41] and Cho et al. [42]. Participating mothers were asked to evaluate the social support received from people around them. This scale comprises five subscales differentiated by the nature of the support: emotional support (two items), instrumental support (three items), sociable support (four items), informational support (three items), and practical support (one item). The emotional support scale comprises items concerning emotional comfort acquired by others’ care, encouragement, empathy, and respect (e.g., “When I feel lonely, I can talk to and rely on people around me [e.g., parents, relatives, friends, acquaintances]”). The instrumental support scale comprises items about tangible assistance such as lending money and goods and providing labor (e.g., “People around me [e.g., parents, relatives, friends, acquaintances] lend me money when I need it urgently”). The sociable support scale comprises items related to activities provided by other people that increase a sense of belonging and social bonds (e.g., “I spend leisure time and vacations with people around me [e.g., parents, relatives, friends, acquaintances]”). The informational support scale comprises items about information received from others when necessary (e.g., “People around me [e.g., parents, relatives, friends, acquaintances] give me information when making important decisions, such as buying a house”). The practical support scale comprises one item about the practical assistance provided by others when one needs help with a specific task (e.g., “People around me [e.g., parents, relatives, friends, acquaintances] provide actual assistance when I need help to do something, such as caring my children”). Each item is scored on a five-point scale ranging from *not at all true* (1) to *very true* (5). This study used the total social support scores. The possible score range was 13–65 points, with higher scores indicating greater social support from others. The Cronbach’s α of the overall scale was 0.94: 0.72 for the emotional support subscale, 0.77 for the instrumental support subscale, 0.85 for the sociable support subscale, and 0.85 for the informational subscale.

#### 2.2.3. Perception of Health

Parents’ perceived health was measured by asking participating mothers and fathers about their own state of health. Each item is scored on a five-point scale ranging from *not healthy at all* (1) to *very healthy* (5). Each of the possible score ranges for the perception of the health of parents is 1–5 points; higher scores indicate a greater degree to which parents perceive themselves as healthy.

#### 2.2.4. Marital Satisfaction

Parental marital satisfaction was measured using the scale used in Chung’s study [43], which is a revised version of the Kansas Marital Satisfaction Scale [44]. Using this scale, each parent evaluates their marital satisfaction. This scale comprises four items, each scored on a five-point scale ranging from *very dissatisfied* (1) to *very satisfied* (5). The possible score range is 4–20 points; higher scores on the scale indicate higher marital satisfaction as perceived by parents. The Cronbach’s α of the marital satisfaction scale of fathers and mothers was 0.94 and 0.93, respectively.

#### 2.2.5. Parental Warmth and Control

Parental warmth and control were measured using the scales developed by the PSKC research team based on Cho et al.’s [45] Korean Parenting Style Scale. Each participating parent evaluated the parental warmth and control they provided while raising their children. The parental warmth scale comprises six items for measuring the degree to which parents show love and concern for their children, act in positive ways toward them, and support their individuality by sensitively responding to their needs and opinions (e.g., “I value my child’s opinions and allow my child to express their opinions”). The parental control scale comprises six items to evaluate the degree to which parents establish rules and guidelines for their children’s behaviors and control misbehaviors (e.g., “I set rules and regulations to be followed and make sure my child follows them”). Each item is rated on a five-point scale ranging from *not at all true* (1) to *very true* (5). Each of the possible score ranges for the parental warmth and control scales is 6–30 points, with higher scores indicating a greater degree of parental warmth and control. The Cronbach’s α was 0.89 and 0.86 for the parental warmth scale of fathers and mothers and 0.80 and 0.74 for the parental control scale of fathers and mothers, respectively.

#### 2.2.6. Behavioral Problems

Children’s behavioral problems were assessed using externalizing and internalizing problems. To measure children’s externalizing and internalizing problems, the Korean Version of the Child Behavior Checklist 6–18 Scale (K-CBCL 6–18), originally developed by Achenbach and Rescorla [46] and adapted and standardized by Oh and Kim [47], was used. Specifically, participating mothers evaluated their children’s externalizing and internalizing problems by responding to the externalizing and internalizing problem scales of the K-CBCL 6–18. Externalizing problems, defined as behavioral problems that are expressed externally due to lack of control, include two subscales: aggressive behavior (18 items; e.g., “My child destroys things belonging to his/her family or others”) and rule-breaking behavior (17 items; e.g., “My child breaks rules at home, school, or elsewhere”). Internalizing problems, representing emotional problems that occur internally from excessive control, contain three subscales: anxious/depressed (13 items; e.g., “My child feels he/she has to be perfect”), withdrawn/depressed (8 items; e.g., “My child is unhappy, sad, or depressed”), and somatic complaints (11 items; e.g., “My child is constipated and doesn’t move bowels”). Each item is scored on a three-point scale from *not true* (0) to *very true or often true* (2). Higher scores represented a greater degree of externalizing and internalizing problems. The Cronbach’s α was 0.86 for the externalizing problem scale and 0.81 for the internalizing problem scale.

Measurement information for the study variables is summarized in Appendix A.

### 2.3. Statistical Analysis

Data were analyzed using IBM SPSS Statistics (version 26.0, IBM Co., Armonk, NY, USA) and Mplus (version 8) [48]. First, participants’ demographic characteristics and descriptive properties of the measured variables were analyzed based on frequencies, means, standard deviations, and Pearson’s correlation coefficients. To identify the work–family interface profiles of dual-earner parents based on the combination of WFE and WFC experiences (RQ1), explore social and economic factors (education, household income, number of children, employment status, and social support) influencing the work–family interface profile membership (RQ2), and examine the differences in parent- and child-related outcomes (parents’ perception of health, marital satisfaction, parental warmth/control, and children’s behavioral problems) according to work–family interface profiles (RQ3), an LPA was conducted. As a typical person-centered analytical approach, LPA is a categorical latent variable modeling approach that focuses on identifying discrete subgroups/profiles of individuals who share similar response patterns in measured continuous variables (often called LPA indicators) [49]. LPA assumes that individuals can be categorized, with varying degrees of probabilities, into subpopulations that have different configurable profiles of psychosocial attributes.

This study adopted an automatic three-step method within Mplus. First, the method determined the number of latent profiles based on indicators (levels of WFE and WFC in fathers and mothers in this study). Second, the most likely profile membership of participants was confirmed based on the posterior probability obtained in the first step. Third, this method identified the associations between latent profiles and external variables (social and economic factors influencing the work–family interface profile membership and outcome variables in this study). The three-step method is beneficial because it keeps the profile classification uninfluenced by external variables while estimating the relationships between the latent profiles and external variables [50].

To determine the optimal number of work–family interface profiles in the first step, several fit statistics, the proportion of members in the derived profiles, and posterior probability were sequentially examined [49]. First, this study started with a two-profile model and then increased the number of profiles by one, comparing the model fit statistics with the previous profile. The Akaike’s information criterion (AIC), Bayesian information criterion (BIC), and sample-size adjusted BIC (SABIC) were used to select the optimal number of latent profiles [51], with lower AIC, BIC, and SABIC values indicating a better profile solution. In addition, the Lo–Mendell–Rubin adjusted likelihood ratio test (LRT) and bootstrap likelihood ratio test (BLRT) were examined; each was accompanied by a *p*-value for a comparison of model fit with one less profile, in which a significant *p*-value (*p* < 0.05) indicated that the K profiles solution provides better fit to the observed data than the K-1 profiles solution [49,51]. The entropy index was also used to evaluate the classification accuracy; scores ranged from 0 to 1, with a higher value representing greater accuracy [49].

The proportion of members in each profile was also examined. Any profile with less than 5% members in the sample is typically considered spurious and can fail to replicate in independent datasets [52]. Therefore, this profile was rejected. Finally, the posterior probability, referred to as the average probability of a person being assigned to a profile given their scores on LPA indicators, was used to further validate the classification, with a value ≥0.80 representing good discriminability [53].

After determining the profile classification, a one-way analysis of variance (ANOVA) was conducted to test whether the profiles generated by LPA were significantly different from one another. Bonferroni post hoc tests were used to determine which specific profiles differed significantly from one another. Finally, each profile is given a name that best describes its characteristics, differentiating it from other profiles; in particular, this study adopted the terminology of work–family interface profiles used in Rantanen et al.’s studies [16,18], as the terminology has been employed in many studies [7,19].

Subsequently, to identify which social and economic factors (education, household income, number of children, employment status, and social support) influence the work–family interface profile membership (RQ2), this study performed a logistic regression analysis using the R3STEP command in LPA, an effective method for verifying the antecedents of latent profile classification [54]. Further, the automatic Bolck–Croon–Hagenaars (BCH) procedure was utilized to verify the differences in the outcome variables (parents’ perception of health, marital satisfaction, parental warmth/control, and children’s behavioral problems) among the latent profiles (RQ3). The BCH approach allows for classification errors and unequal variance across profiles, which blocks the possibility of changes in latent profiles and enables accurate comparisons between profiles [54].

## 3. Results

### 3.1. Descriptive Statistics and Correlations

Table 3 presents the means and standard deviations of the variables. First, the average WFC scores were low in fathers (*M* = 2.41, *SD* = 0.59) and mothers (*M* = 2.74, *SD* = 0.66), whereas the mean WFE scores (five-point scale) in fathers (*M* = 3.73, *SD* = 0.57) and mothers (*M* = 3.63, *SD* = 0.52) were relatively high. The average score for social support was also relatively high at 3.90 (*SD* = 0.57) out of a possible score of five. The mean scores for perceptions of health in fathers (*M* = 3.38, *SD* = 0.74) and mothers (*M* = 3.36, *SD* = 0.79) were above the midpoint of the five-point scoring range. In addition, the average marital satisfaction was high for fathers (*M* = 4.04, *SD* = 0.70) and mothers (*M* = 3.71, *SD* = 0.80), with a possible score of five. Further, the means of paternal and maternal warmth (five-point scale) were relatively high at 3.61 (*SD* = 0.62) and 3.72 (*SD* = 0.56), respectively, whereas the average scores of paternal (*M* = 3.41, *SD* = 0.58) and maternal (*M* = 3.55, *SD* = 0.50) control were also above the midpoint of the possible score of five. The average scores for externalizing and internalizing problems were 4.27 (*SD* = 4.45) and 3.59 (*SD* = 3.90), respectively.

Additionally, paired *t*-tests were conducted to identify differences in WFE, WFC, perception of health, marital satisfaction, and parental warmth and control according to parental sex. The results indicated that fathers’ WFE (*t* = 3.62, *p* < 0.001) and marital satisfaction (*t* = 10.12, *p* < 0.001) were significantly higher than the corresponding scores of mothers. However, mothers’ scores of WFC (*t* = 9.36, *p* < 0.001), parental warmth (*t* = 3.61, *p* < 0.001), and parental control (*t* = 5.22, *p* < 0.001) were higher than the corresponding scores of fathers.

The correlations among the study variables are presented in Appendix B. While both parents’ WFE was positively correlated with social support, WFC was negatively correlated with social support. Fathers’ WFE (or WFC) positively (or negatively) correlated with the health of both parents; however, mothers’ WFE (or WFC) positively (or negatively) correlated with their own health alone. In addition, both parents’ WFE (or WFC) positively (or negatively) correlated with their own and the other parents’ marital satisfaction, and both parents’ WFE positively correlated with their own and the other parents’ warmth. Fathers’ WFC was negatively correlated with both parents’ warmth, whereas mothers’ WFC was negatively correlated with their own parental warmth. Fathers’ WFE alone positively correlated with their own parental control. Mothers’ WFE (or WFC) was positively (or negatively) correlated with children’s behavioral problems, whereas fathers’ WFC alone was positively correlated with children’s behavioral problems.

### 3.2. Classification of Work–Family Interface Profiles

To determine the number of work–family interface profiles for dual-income parents (RQ1), the fit statistics of the two to five latent profile models presented in Table 4 were compared. All values of AIC, BIC, and SABIC decreased with each increase in the number of latent profiles from two to four; however, the BIC value increased in the five-profile model compared with the four-profile model. In addition, the BLRT showed significant results in all comparisons of model fit between the K and K-1 profile models. However, because the *p*-value of the LRT was 0.491 in the five-profile model, the profile model was rejected. Moreover, the four-profile model was excluded because it produced a profile with a portion of members equivalent to less than 5% of the sample, the threshold suggested by Berlin et al. [52]. The three-profile model has good fit indices and acceptable proportions of members in the profiles. Additionally, most posterior probabilities were above or close to 0.80. Therefore, the three-profile model was selected as the best classification model for describing the data.

The results of the ANOVA performed to verify the mean differences in parental WFE and WFC among the three latent profiles are presented in Table 5. The means of paternal WFE in Profiles 1 and 2 were significantly higher than the corresponding score in Profile 3, whereas the averages of paternal WFC in Profiles 1 and 2 were lower than the corresponding score in Profile 3. However, no significant differences were found in the means of paternal WFE and WFC between Profiles 1 and 2. However, the mean scores of both maternal WFE and WFC in Profile 1 were between the corresponding values of Profiles 2 and 3. The means of both parents’ WFE in Profile 2 were the highest, whereas the averages of both parents’ WFC were the lowest. The average scores of both parents’ WFC in Profile 3 were the highest, with the means of both parents’ WFE being the lowest.

Therefore, by adopting the terminology of work–family interface profiles in Rantanen et al.’s studies [16,18], Profile 1 was named *Beneficial fathers/Moderate active mothers*, as the fathers’ WFE level was high, while the WFC level was low, and the mothers’ WFE and WFC were both intermediate levels and were close to their respective overall averages, as presented in Table 3. In addition, based on the characteristics of the father’s and mother’s work–family interfaces described above, Profile 2 was labeled *Beneficial* and Profile 3 was labeled *Harmful*.

### 3.3. Influences of Social and Economic Variables on the Work–Family Interface Profile Membership

Using the R3STEP command, a logistic regression analysis was conducted to identify which social and economic variables influence the work–family interface profile membership. Table 6 presents the results. Fathers’ education and household income influenced the work–family interface profile membership. Specifically, fathers with higher education levels had a significantly higher likelihood of belonging to the *Beneficial fathers/Moderate active mothers* profile than to the *Harmful* profile (B = 0.48, *p* < 0.01, OR = 1.63). In addition, the greater the household income, the higher the likelihood of belonging to the *Beneficial fathers/Moderate active mothers* profile than to the *Harmful* profile (B = 1.01, *p* < 0.05, OR = 2.76). Social support was also associated with the work–family interface profile membership. The higher the social support, the higher the likelihood of belonging to the *Beneficial* profile than to the *Beneficial fathers/Moderate active mothers* profile (B = −1.89, *p* < 0.001, OR = 0.15); of belonging to the *Beneficial fathers/Moderate active mothers* profile than to the *Harmful* profile (B = 0.68, *p* < 0.05, OR = 1.98); and of belonging to the *Beneficial* profile than to the *Harmful* profile (B = 2.58, *p* < 0.001, OR = 13.13).

### 3.4. Differences in Parent- and Child-Related Outcomes among the Work–Family Interface Profiles

To examine the differences in parent- and child-related outcomes among the work–family interface profiles, the BCH command was used. As presented in Table 7, significant differences were found in the perception of health, marital satisfaction, paternal and maternal warmth and control, and children’s behavioral problems among the three work–family interface profiles. Specifically, fathers in the *Beneficial fathers/Moderate active mothers* and *Beneficial* profiles evaluated themselves as healthier than fathers in the *Harmful* profile (χ^2^ = 41.448, *p* < 0.001). Moreover, mothers in the *Beneficial fathers/Moderate active mothers* and *Beneficial* profiles perceived themselves to be healthier than those in the *Harmful* profile, with mothers in the *Beneficial* profile regarding themselves to be healthier than those in the *Beneficial fathers/Moderate active mothers* profile (χ^2^ = 21.644, *p* < 0.001). The marital satisfaction for both parents in the *Beneficial fathers/Moderate active mothers* and *Beneficial* profiles were significantly higher than those for both parents in the *Harmful* profile (fathers: χ^2^ = 69.973, *p* < 0.001; mothers: χ^2^ = 68.911, *p* < 0.001).

The parental warmth for both parents in the *Beneficial fathers/Moderate active mothers* and *Beneficial* profiles were significantly higher than those for both parents in the *Harmful* profile (fathers: χ^2^ = 120.302, *p* < 0.001; mothers: χ^2^ = 47.392, *p* < 0.001). Further, the parental control for both parents in the *Beneficial fathers/Moderate active mothers* profile was higher than those for both parents in the *Harmful* profile (fathers: χ^2^ = 16.072, *p* < 0.001; mothers: χ^2^ = 6.789, *p* < 0.05). The degrees of externalizing and internalizing problems in the children of the *Harmful* profile were greater than those of the corresponding problems in the children of the *Beneficial fathers/Moderate active mothers* and *Beneficial* profiles (externalizing problems: χ^2^ = 13.962, *p* < 0.01; internalizing problems: χ^2^ = 13.103, *p* < 0.01).

## 4. Discussion

This study identified the couple-level profiles of the work–family interface among South Korean dual-income parents and the influences of social and economic factors on the work–family interface profile membership. It also examined the differences in parent- and child-related outcomes among the work–family interface profiles.

First, the LPA revealed three profiles showing different levels of WFE and WFC among dual-income parents. Of the four distinct types of work–family interface that emerged in prior research [16,18,19], two profiles (*Beneficial* and *Harmful* profiles—both of which emerged as similar interface patterns for both parents) were identified. However, the remaining profile was *Beneficial fathers/Moderate active mothers*, a dissimilar interface pattern for husbands and wives. This profile has not been reported in previous studies that analyzed the work–family interface of dual-income parents at the couple level in Western and Eastern cultures [3,26]. In addition, the *Beneficial fathers/Moderate active mothers* and *Harmful* profiles were the most prevalent, whereas the *Beneficial* profile showed the lowest prevalence. These results are partially consistent with those of recent studies conducted in East Asia, such as China and South Korea [21,26], wherein the *Conflictual* type, similar to the *Harmful* profile, was the most prevalent one. However, the *Beneficial* type has been reported to be dominant in many studies conducted in Western culture [3,19,20]. The average annual working hours per worker in South Korea are much higher than those in the US and France [22]. Moreover, approximately 70% of South Korean workers experience WFC because of overtime, night work, and job instability [11]. Therefore, the current findings (i.e., the dominant prevalence of *Harmful* profile) suggest the need to develop effective policy support to improve work–family balance at the national and corporate levels in South Korea. Further, the *Beneficial fathers/Moderate active mothers* profile, along with the *Harmful* profile, was the largest group identified. In particular, when fathers belonged to the *Beneficial* profile, mothers were more likely to show the *Moderately active* profile than the *Beneficial* one. Such a result indicates that in many cases, paternal WFE is not always positively transmitted to mothers, as posited by family systems theory. Women are primarily responsible for childcare and housework in many societies [3]. Moreover, in the Confucian collectivistic cultures prevalent in South Korea and China, the boundaries between work and family are often blurred, as work is often seen as a means to advance a family’s economic status [24,25]. Therefore, working mothers in South Korea may have higher WFE and WFC because they are highly engaged in work and family roles.

In addition, the results in Jung et al.’s study [26] using the same data (PSKC) as this study showed that, unlike in this study, the *Beneficial fathers/Harmful mothers* profile was reported as the dominant type. The gap in these research results may be explained by differences in research methods. Specifically, while this study measured the family domain of the work–family interface as a comprehensive environment in which family life and child-rearing co-occur, Jung et al. [26] divided the family domain into childcare and family life and analyzed the work–family interface profiles by categorizing the enrichment and conflict between work and childcare and between work and family life into four types. In addition, when classifying the work–family interface profiles, Jung et al. [26] tend to overestimate or underestimate the levels of WFC or WFE by focusing on comparing the averages of profiles while dismissing the overall average levels of WFC and WFE. Therefore, as researchers have adopted different criteria for classifying work–family interface types, it seems necessary to establish a consistent basis for these criteria.

Second, this study showed that fathers’ education level, household income, and social support influenced the work–family interface profile membership; specifically, the higher the fathers’ education level, household income, and social support, the more likely it was that the dual-income parents belonged to the *Beneficial fathers/Moderate active mothers* profile rather than to the *Harmful* profile. In addition, the higher the social support, the higher the likelihood of belonging to the *Beneficial* profile than to the *Beneficial fathers/Moderate active mothers* profile. These results are consistent with those of prior studies [26,28,29], wherein the higher the household income and social support, the higher the WFE and the lower the WFC. Income and social support are recognized as major factors promoting the WFE [28,29]. A high income can satisfy the demands of both employees and their families. In addition, individuals with high household incomes are typically in high-income occupations or have spouses in high-income occupations. High-income occupations are more likely to have formal, family-friendly benefits than low-income occupations, and these benefits are often accessible and/or useful to both employees and spouses [29,55].

Higher educational attainment leads to more attractive job opportunities, greater labor force flexibility, and more rewarding jobs [56,57]. However, while mothers’ education was not associated with the work–family interface profile membership, social support was the only factor determining whether dual-earner parents, especially mothers, were members of the *Beneficial* profile. This result may be explained by the findings of Solomon et al. [58], wherein the negative relationship between women’s education levels and job satisfaction was stronger than that of men. Solomon et al. [58] argued that highly educated women are more likely to earn higher income, but they experience significantly less autonomy, greater qualitative demands at work, and more hours worked than their male counterparts. Given that women hold primary responsibility for childcare and housework in many societies [3], highly educated women may shoulder greater responsibility in the household and labor markets to adhere to gender role expectations while advancing in their careers [58]. Therefore, social support may play a key role in reducing WFC and increasing WFE in women.

Third, higher parent- and child-related outcomes were reported in the *Beneficial fathers*/*Moderate active mothers* and *Beneficial* profiles than in the *Harmful* profile. Specifically, while the perception of health, marital satisfaction, and parental warmth were higher in the *Beneficial fathers/Moderate active mothers* and *Beneficial* profiles than in the *Harmful* profile, children’s behavioral problems were higher in the *Harmful* profile than in the other two profiles. In particular, mothers in the *Beneficial* profile perceived their health to be the highest compared to the other two profiles.

These results are compatible with previous studies reporting that the *Beneficial* group had better health perception and higher life satisfaction, while the *Harmful* group showed poorer health perception and lower life satisfaction [3,7,16]. In addition, consistent with the results of Vieira et al. [3], indicating that the *Active* and *Beneficial* groups reported higher family satisfaction than the *Harmful* group, both the *Beneficial* and *Beneficial fathers/Moderate active mothers* profiles showed higher marital satisfaction and children’s psychological health compared to the *Harmful* profile in this study. These results suggest that situations in which fathers’ WFE is high and mothers’ active participation in home and work life do not have a significant and negative impact on family relationships; in particular, for the *Active* group, higher investment in work–family roles may lead to higher WFC and WFE, but WFE experience may offset the negative impact of WFC to some extent. Therefore, the current results generally support work–family spillover and crossover processes.

In addition, parental control was reported to be higher in the *Beneficial fathers/Moderate active mothers* profile than in the *Harmful* profile. This finding supports the results of several studies [59,60] that reported positive relationships between parental WFC levels and low involvement, poor monitoring and supervision, and neglect. This can be explained using the scarcity hypothesis [9]: Parents who experience high WFC may not have the time and energy to devote themselves to their families and thus may be more likely to give insufficient control or supervision to their children. Moreover, in many countries, mothers are still regarded as primarily responsible for raising children and doing housework [3]. *Active* mothers, who tend to be highly engaged in their roles at work and home [18], may be more likely to impose their children’s responsibilities on their behaviors by setting more controls and rules together with fathers to manage their family life and children more effectively.

The findings have practical implications for theory and practice. Traditionally, some scholars considered the work–family interface as a single continuum with positive and negative sides [15]. However, the current results support the theoretical argument that WFE and WFC represent two different types characterized by an orthogonal relationship and manifest in many forms that result from the combinations of the two types. Regarding clinical practice, this study recommends adopting a couple-based approach to intervention programs and tailored intervention strategies to fit the work–family interface types in terms of effectiveness. The study’s results also indicate that in many cases, the wives belonged to the *Moderate active* type with moderate WFC and WFE, even when the husbands were members of the *Beneficial* group. In particular, given that the higher the social support, the higher the likelihood of belonging to the *Beneficial* profile than to the *Beneficial fathers/Moderate active mothers* or *Harmful* profiles, policymakers and human resource managers should provide various supportive strategies for their employees, particularly for women. Especially if WFC cannot be avoided, its negative effects on individual workers should be offset by the development of WFE. WFE is facilitated by the work–family balance support systems [11]. Organizations frequently adhere to taken-for-granted policies and practices, such as mandatory early morning or late afternoon business meetings, that undermine organizational performance and interfere with workers’ ability to balance work and family [8]. One possible support might be to introduce policies that give workers more autonomy in how they manage work and home responsibilities, such as allowing workers to determine the time and methods to better manage work and family activities [19]. Lengthening paid parental leave and expanding afterschool public care systems should also be considered at the national level. Additionally, career counselors should recognize the negative outcomes of the *Harmful* profile, as the present study identified. Especially when counseling employees on work–family issues, it might be useful to encourage more efficient work recovery processes (e.g., time management techniques, self-reflection) to protect employees’ well-being and facilitate positive spillover between their work and personal roles [61]. Additionally, considering that a majority of the women participating in this study were found to be members of the *Moderate active* type, it is important to discuss the danger of having excessively blurred boundaries between the work and family domains in counseling, not being able to separate work and other important aspects of life and being accessible at all times mean constant activation, reducing the time available for rest and recovery [16].

Considering its limitations, this study offers several directions for future research. First, it mainly relied on parents’ evaluations of WFE, WFC, and socioeconomic and outcome variables; thus, the associations observed in this study may have been overestimated because of common-method variance. At the very least, future studies could use multiple informants (e.g., teachers) to collect data on children’s behavioral problems. Second, this study did not consider the directionality of work and family in the work–family interface (work–family or family–work). However, as different types of work–family interfaces are produced depending on the source of stress or resources (e.g., work-to-family conflict or enrichment, family-to-work conflict or enrichment; Vieira et al. [3]), further research is needed to analyze the work–family interfaces by considering directionalities. Finally, a longitudinal study of the couple-based work–family interface is needed to clearly identify the influence of antecedent factors of the work–family interface. In addition, future studies, unlike this study, need to comprehensively examine how family and work outcomes differ depending on the type of work–family interface by adding work-related factors (e.g., job productivity) as outcome variables.

## 5. Conclusions

Based on a person-centered approach, this study identified three differential work–family interface profiles (*Beneficial fathers/Moderate active mothers*, *Beneficial*, and *Harmful*) among South Korean double-income couples at a dyadic level. In addition, fathers’ education, household income, and social support influenced the membership of work–family interface profiles. Working parents belonging to the *Beneficial fathers/Moderate active mothers* and *Beneficial* profiles showed higher health, marital satisfaction, and warm parenting, and their children had lower behavioral problems than those in the *Harmful* profile. Finally, this study recommends that the dyadic-level typological approach to the work–family interface of dual-earner parents is conducive to increasing the effectiveness of interventions by identifying the profiles and their relationships with social and personal factors.

## Figures and Tables

**Table 1:** Typology of work–family interface proposed by Grzywacz et al. [17] and Rantanen et al. [18].

Work–Family Enrichment	Work–Family Conflict	
	High	Low
High	*Blurred* (or *Active*)	*Balanced* (or *Beneficial*)
Low	*Imbalanced* (or *Harmful*)	*Segmented* (or *Passive*)

**Table 2:** Participants’ demographics (*N* = 558).

	Background Characteristics		Frequency (%)	
Fathers	Final educational attainment	High school graduation or lower	141	(25.2)
		Second- or third-year college graduation	118	(21.1)
		University graduation or higher	299	(53.6)
	Employment status	Full-time worker	386	(69.2)
		Temporary worker	9	(1.6)
		Daily worker	11	(2.0)
		Employer with employees	44	(7.9)
		Self-employed without employees	52	(9.3)
		Unpaid family worker	3	(0.5)
		No response	53	(9.5)
Mothers	Final educational attainment	High school graduation or lower	135	(24.2)
		Second- or third-year college graduation	153	(27.4)
		University graduation or higher	270	(48.4)
	Employment status	Full-time worker	356	(63.8)
		Temporary worker	65	(11.6)
		Daily worker	7	(1.3)
		Employer with employees	31	(5.6)
		Self-employed without employees	69	(12.4)
		Unpaid family worker	30	(5.4)
	Monthly household income	Under 2 million	8	(1.4)
		Between 2 and 3.99 million	101	(18.1)
		Between 4 and 5.99 million	262	(47.0)
		Above 6 million	187	(33.5)
	Number of children	One	60	(10.8)
		Two	366	(65.6)
		Three or more	132	(23.6)

**Table 3:** Descriptive statistics of research variables (*N* = 558).

Variables		*M*	*SD*
Work–family enrichment	Fathers	3.73	0.57
	Mothers	3.63	0.52
Work–family conflict	Fathers	2.41	0.59
	Mothers	2.74	0.66
Social support		3.90	0.57
Perception of health	Fathers	3.38	0.74
	Mothers	3.36	0.79
Marital satisfaction	Fathers	4.04	0.70
	Mothers	3.71	0.80
Parental warmth	Fathers	3.61	0.62
	Mothers	3.72	0.56
Parental control	Fathers	3.41	0.58
	Mothers	3.55	0.50
Externalizing problems		4.27	4.45
Internalizing problems		3.59	3.90

**Table 4:** Model fit indices for the compared latent profiles.

No. of Profile	AIC	BIC	SABIC	LRT (*p*)	BLRT (*p*)	Entropy	Smallest Proportion of Members (%)	Posterior Probability				
								1	2	3	4	5
2	3815.797	3872.014	3830.746	0.033 *	0.000 ***	0.616	32.3%	0.875	0.796			
**3**	**3785.933**	**3863.772**	**3806.631**	0.022 *	0.000 ***	**0.691**	**5.8%**	**0.798**	**0.852**	**0.837**		
4	3752.231	3851.692	3778.679	0.005 **	0.000 ***	0.643	4.3%	0.882	0.796	0.795	0.775	
5	3738.306	3859.388	3770.503	0.491	0.000 ***	0.706	0.7%	0.814	0.814	0.886	0.807	0.765

Note: * *p* < 0.05; ** *p* < 0.01; *** *p* < 0.001.

**Table 5:** Differences in parents’ work–family interfaces among the three latent profiles.

Variables	Profile 1 (a) (*n* = 263, 47.1%)	Profile 2 (b) (*n* = 32, 5.8%)	Profile 3 (c) (*n* = 263, 47.1%)	*F*	Bonferroni Post hoc Test
	*M* (S.E.)	*M* (S.E.)	*M* (S.E.)		
Work–family enrichment (father)	4.07 (0.39)	4.20 (0.11)	3.41 (0.09)	215.527 ***	a, b > c
Work–family conflict (father)	2.09 (0.43)	2.14 (0.12)	2.74 (0.08)	192.457 ***	c > a, b
Work–family enrichment (mother)	3.65 (0.08)	4.42 (0.20)	3.46 (0.04)	100.000 ***	b > a, c; a > c
Work–family conflict (mother)	2.69 (0.09)	1.77 (0.18)	2.93 (0.05)	77.704 ***	c > a, b; a > b

Note: Profile 1 = *Beneficial fathers/Moderate active mothers*; Profile 2 = *Beneficial*; Profile 3 = *Harmful. M* = mean; S.E. = standard error. *** *p* < 0.001.

**Table 6:** Influences of social and economic factors on the work–family interface profile membership.

Variables	Profile 1 (*Beneficial Fathers/Moderate Active Mothers*) vs. Profile 2 (*Beneficial*; R)		Profile 1 (*Beneficial Fathers/Moderate Active Mothers*) vs. Profile 3 (*Harmful*; R)		Profile 2 (*Beneficial*) vs. Profile 3 (*Harmful*; R)	
	B (S.E.)	OR	B (S.E.)	OR	B (S.E.)	OR
Education (father)	0.12 (0.32)	1.13	0.48 (0.16) **	1.63	0.36 (0.28)	1.44
Education (mother)	−0.16 (0.31)	0.85	0.28 (0.16)	1.32	0.44 (0.28)	1.55
Household income (log)	0.57 (0.88)	1.77	1.01 (0.46) *	2.76	0.44 (0.73)	1.56
Number of children	−0.22 (0.32)	0.80	−0.21 (0.23)	0.81	0.01 (0.28)	1.01
Employment status (father)	−0.54 (0.65)	1.72	−0.58 (0.35)	1.79	−0.04 (0.61)	1.03
Employment status (mother)	−0.06 (0.52)	1.06	0.07 (0.29)	0.93	0.13 (0.47)	0.88
Social support	−1.89 (0.50) ***	0.15	0.68 (0.31) *	1.98	2.58 (0.48) ***	13.13

Note: R = reference group; B = regression coefficient; S.E. = standard error; OR = odds ratio. * *p* < 0.05; ** *p* < 0.01; *** *p* < 0.001.

**Table 7:** Differences in parent- and child-related outcomes among the three latent profiles.

Outcomes	Profile 1 (*Beneficial Fathers/Moderate Active Mothers*; a)	Profile 2 (*Beneficial*; b)	Profile 3 (*Harmful*; c)	Overall Chi-Squared Test
	*M* (S.E.)	*M* (S.E.)	*M* (S.E.)	
Perception of health (fathers)	3.67 (0.06)	3.59 (0.17)	3.08 (0.06)	41.448 *** (a, b > c)
Perception of health (mothers)	3.47 (0.06)	3.84 (0.15)	3.18 (0.06)	21.644 *** (a, b > c; b > a)
Marital satisfaction (fathers)	4.29 (0.06)	4.54 (0.10)	3.72 (0.06)	69.973 *** (a, b > c)
Marital satisfaction (mothers)	4.04 (0.06)	4.22 (0.13)	3.32 (0.07)	68.911 *** (a, b > c)
Paternal warmth	3.97 (0.05)	4.03 (0.13)	3.22 (0.05)	120.302 *** (a, b > c)
Paternal control	3.57 (0.05)	3.43 (0.13)	3.26 (0.04)	16.072 *** (a > c)
Maternal warmth	3.89 (0.05)	4.12 (0.11)	3.50 (0.04)	47.392 *** (a, b > c)
Maternal control	3.64 (0.04)	3.57 (0.14)	3.47 (0.04)	6.789 * (a > c)
Externalizing problems (children)	3.41 (0.36)	2.89 (0.76)	5.28 (0.38)	13.962 ** (c > a, b)
Internalizing problems (children)	3.14 (0.31)	2.04 (0.55)	4.26 (0.34)	13.103 ** (c > a, b)

Note: *M* = mean; S.E. = standard error. * *p* < 0.05; ** *p* < 0.01; *** *p* < 0.001.

## Data Availability

This study was conducted using publicly available data (https://panel.kicce.re.kr/pskc/module/rawDataManage/index.do?menu_idx=56; accessed on 16 May 2024).

## Appendix A

**Table A1:** Information of Measurement for the Research Variables.

Concept	Scale Information	Response Options
Final education attainment	Nominal scale	*no education* (1), *elementary school graduation* (2), *middle school graduation* (3), *high school graduation* (4), *second- or third-year college graduation* (5), *university graduation* (6), *graduate school graduation* (7)
Employment status	Nominal scale	*full-time worker* (1), *temporary worker* (2), *daily worker* (3), *employer with employees* (4), *self-employed without employees* (5), *unpaid family worker* (6)
Household income	-	Open-ended
Number of children	-	Open-ended
Work–family interface	5-point Likert scale	*not at all true* (1), *slightly true* (2), *moderately true* (3), *quite a bit true* (4), *very true* (5)
Social support	5-point Likert scale	*not at all true* (1), *slightly true* (2), *moderately true* (3), *quite a bit true* (4), *very true* (5)
Perception of health	5-point Likert scale	*not healthy at all* (1), *slightly healthy* (2), *moderately healthy* (3), *quite a bit healthy* (4), *very healthy* (5)
Marital satisfaction	5-point Likert scale	*very dissatisfied* (1), *quite a bit dissatisfied* (2), *moderately satisfied* (3), *quite a bit satisfied* (4), *very satisfied* (5)
Parental warmth and control	5-point Likert scale	*not at all true* (1), *slightly true* (2), *moderately true* (3), *quite a bit true* (4), *very true* (5)
Behavioral problems	3-point Likert scale	*not true* (0), *somewhat or sometimes true* (1), *very true or often true* (2)

## Appendix B

**Table A2:** Correlations between the Study Variables.

	1	2	3	4	5	6	7	8	9	10	11	12	13	14
1														
2	−0.38 **													
3	0.18 **	−0.12 **												
4	−0.11 **	0.12 **	−0.33 **											
5	0.12 **	−0.14 **	0.32 **	−0.16 **										
6	0.24 **	−0.22 **	0.05	−0.06	0.07									
7	0.12 **	−0.18 **	0.16 **	−0.14 **	0.09 *	0.14 **								
8	0.33 **	−0.29 **	0.11 **	−0.11 **	0.14 **	0.28 **	0.12 **							
9	0.28 **	−0.25 **	0.21 *	−0.22 **	0.15 **	0.25 **	0.26 **	0.48 **						
10	0.51 **	−0.31 **	0.14 **	−0.08	0.15 **	0.23 **	0.11 **	0.39 **	0.39 **					
11	0.18 **	−0.06	0.05	−0.05	0.08 *	0.04	0.05	0.16 **	0.12 **	0.27 **				
12	0.22 **	−0.18 **	0.27 **	−0.15 **	0.15 **	0.02	0.15 **	0.25 **	0.32 **	0.34 **	0.04			
13	0.08	−0.01	0.08	0.04	0.07	−0.03	0.01	0.12 **	0.20 **	0.08	0.25 **	0.21 **		
14	−0.07	0.11 **	−0.11 **	0.15 **	−0.03	0.04	−0.14 **	−0.10 *	−0.22 **	−0.10 *	−0.01	−0.30 **	−0.03	
15	−0.04	0.18 **	−0.11 **	0.15 **	−0.10 *	0.02	−0.17 **	−0.11 **	−0.16 **	−0.07	0.01	−0.23 **	−0.06	0.63 **

Note: 1 = work–family enrichment (father); 2 = work–family conflict (father); 3 = work–family enrichment (mother); 4 = work–family conflict (mother); 5 = social support; 6 = perception of health (father); 7 = perception of health (mother); 8 = marital satisfaction (father); 9 = marital satisfaction (mother); 10 = paternal warmth; 11 = paternal control; 12 = maternal warmth; 13 = maternal control; 14 = externalizing problems; 15 = internalizing problems. * *p* < 0.05; ** *p* < 0.01.

## References

[1] Frone M.R., Yardley J.K., Markel K.S. (1997). Developing and testing an integrative model of the work–family interface. J. Vocat. Behav..

[2] Ford M., Wang Y.-R., Huh Y., Diener E., Oishi S., Tay L. (2017). Work, the work–family interface, and subjective well-being. Handbook of Well-Being.

[3] Vieira J.M., Matias M., Lopez F.G., Matos P.M. (2018). Work–family conflict and enrichment: An exploration of dyadic typologies of work–family balance. J. Vocat. Behav..

[4] Howard M.C., Hoffman M.E. (2018). Variable-centered, person-centered, and person-specific approaches: Where theory meets the method. Organ. Res. Methods.

[5] Kwon J.-K. (2014). Analysis on the roles of group bullying in elementary school—Profile exploration, prediction and longitudinal change analysis. J. Korean Soc. Child Welf..

[6] Lim Y. (2024). Parents’ work–family conflict and children’s behavioral problems: Mediating roles of parental warmth and children’s executive function difficulties. Curr. Psychol..

[7] Yucel D. (2021). Different types of work–family balance, social support, and job satisfaction: A latent class analysis. Appl. Res. Qual. Life.

[8] Grzywacz J.G., Carlson D.S. (2007). Conceptualizing work–family balance: Implications for practice and research. Adv. Dev. Hum. Resour..

[9] Goode W.J. (1960). A theory of role strain. Am. Sociol. Rev..

[10] Kahn R.L., Wolfe D.M., Quinn R.P., Snoek J.D., Rosenthal R.A. (1964). Organizational Stress: Studies in Role Conflict and Ambiguity.

[11] Kim W. (2018). Seven out of 10 People Experience Conflict between Home and Work…Difficulty Reconciling Work and Family. Newsfreezone.

[12] Sieber S.D. (1974). Toward a theory of role accumulation. Am. Sociol. Rev..

[13] Marks S.R. (1977). Multiple roles and role strain: Some notes on human energy, time and commitment. Am. Sociol. Rev..

[14] Greenhaus J.H., Powell G.N. (2006). When work and family are allies: A theory of work–family enrichment. Acad. Manag. Rev..

[15] Frone M.R., Quick J.C., Tetrick L.E. (2003). Work–family balance. Handbook of Occupational Health Psychology.

[16] Rantanen J., Kinnunen U., Mauno S., Tement S. (2013). Patterns of conflict and enrichment in work–family balance: A three-dimensional typology. Work. Stress.

[17] Grzywacz J.G., Butler A.B., Almeida D.M., Marcus-Newhall A., Halpern D.F., Tan S.J. (2008). Work, family, and health: Work–family balance as a protective factor against stresses of daily life. The Changing Realities of Work and Family.

[18] Rantanen J., Kinnunen U., Mauno S., Tillemann K., Kaiser S., Ringlstetter J., Pina e Cunha M., Eikhof D.R. (2011). Introducing theoretical approaches to work-life balance and testing a new typology among professionals. Creating Balance? International Perspectives on the Work-Life Integration of Professionals.

[19] Carvalho V.S., Chambel M.J. (2016). Work-to-family enrichment and conflict profiles: Job characteristics and employees’ well-being. Span. J. Psychol..

[20] Robinson L.D., Magee C.A., Caputi P. (2016). Work-to-family profiles, family structure and burnout in mothers. J. Manag. Psychol..

[21] Zhang C., Dik B.J., Dong Z. (2022). Living a calling and work–family interface: A latent profile analysis. J. Career Assess..

[22] Organisation for Economic Co-operation and Development Average Annual Hours Actually Worked per Worker. https://data-explorer.oecd.org/vis?df[ds]=DisseminateFinalDMZ&df[id]=DSD_HW@DF_AVG_ANN_HRS_WKD&df[ag]=OECD.ELS.SAE.

[23] Organisation for Economic Co-Operation and Development OECD Better-Life Index: Work-Life Balance. https://www.oecdbetterlifeindex.org/topics/work-life-balance/.

[24] Cho E., Choi Y., Shockley K.M., Shen W., Johnson R.C. (2018). A review of work–family research in Confucian Asia. The Cambridge Handbook of the Global Work–Family Interface.

[25] Hofstede G. (2001). Culture’s Consequences: Comparing Values, Behaviors, Institutions, and Organizations Across Nations.

[26] Jung Y.M., Kang H., Oh S.H. (2022). Analysis of parents’ classification of work–family strains and gains and affecting factors. J. Korean Counc. Child. Rights.

[27] Sung M., Ki P. (2021). Children’s happiness and executive function difficulty associated with working mothers’ profiles in parenting behaviors and work–family balance. J. Fam. Better Life.

[28] Lapierre L.M., Li Y., Kwan H.K., Greenhaus J.H., DiRenzo M.S., Shao P. (2018). A meta-analysis of the antecedents of work–family enrichment. J. Organ. Behav..

[29] Ford M.T. (2011). Linking household income and work–family conflict: A moderated mediation study. Stress Health.

[30] Moazami-Goodarzi A., Nurmi J.-E., Mauno S., Aunola K., Rantanen J. (2019). Longitudinal latent profiles of work–family balance: Examination of antecedents and outcomes. Int. J. Stress Manag..

[31] Cooklin A.R., Westrupp E., Strazdins L., Giallo R., Martin A., Nicholson J.M. (2015). Mothers’ work–family conflict and enrichment: Associations with parenting quality and couple relationship. Child Care Health Dev..

[32] Magee C.A., Stefanic N., Caputi P., Iverson D.C. (2012). The association between job demands/control and health in employed parents: The mediating role of work-to-family interference and enhancement. J. Occup. Health Psychol..

[33] Matias M., Recharte J. (2021). Links between work–family conflict, enrichment, and adolescent well-being: Parents’ and children’s perspectives. Fam. Relat..

[34] Yucel D., Latshaw B.A. (2021). How do mothers’ and fathers’ work–family conflict impact children’s problem behaviors?. J. Fam. Issues.

[35] Bakker A.B., Demerouti E., Grzywacz J.G., Demerouti E. (2013). The spillover-crossover model. New Frontiers in Work and Family Research.

[36] Demerouti E., Geurts S. (2004). Towards a typology of work-home interaction. Community Work Fam..

[37] Mauno S., Rantanen J., Kinnunen U., IBOP (2011). Work–family balance and its correlates among Finnish academic professionals: Profiling the experiences of work–family conflict and enrichment. The Future of Knowledge-Intensive Service Work: Theory and Practice of Managing Human and Organizational Resources.

[38] Ku S., Chung I. (2021). Factors affecting the women’s career interruption during children’s elementary school entrance period. Women’s Stud..

[39] Korea Institute of Child Care and Education Panel Study on Korean Children. https://panel.kicce.re.kr/pskc/module/rawDataManage/index.do?menu_idx=56.

[40] Marshall N.L., Barnett R.C. (1993). Work–family strains and gains among two-earner couples. J. Community Psychol..

[41] Lee J.R., Ok S.W. (2001). Family life events, social support, support from children, and life satisfaction of the low-income female earners. Hum. Ecol. Res..

[42] Cho B.E., Suh D.I., Shin H.-Y., Chung H.-S. (1998). The impact of coping resources on positive changes of single mothers and their children. J. Korean Home Econ. Assoc..

[43] Chung H. (2004). Application and revision of the Kansas Marital Satisfaction Scale for use with Korean couples. Psychol. Rep..

[44] Schumm W.R., Nichols C.W., Schectman K.L., Grigsby C.C. (1983). Characteristics of responses to the Kansas Marital Satisfaction Scale by a sample of 84 married mothers. Psychol. Rep..

[45] Cho B., Lee J., Lee H., Kwon H. (1999). Dimensions and assessment of Korean parenting style. Fam. Environ. Res..

[46] Achenbach T.M., Rescorla L.A. (2001). Manual for the ASEB, A School-Age Forms & Profiles.

[47] Oh K., Kim Y. (2010). Manual for Korean Version of the Child Behavior Checklist for Ages 6–18.

[48] Muthén L.K., Muthén B.O. (2017). Mplus: Statistical Analysis with Latent Variables: User’s Guide (Version 8).

[49] Tein J.Y., Coxe S., Cham H. (2013). Statistical power to detect the correct number of classes in latent profile analysis. Struct. Equ. Model..

[50] Asparouhov T., Muthén B. (2014). Auxiliary variables in mixture modeling: Three-step approaches using Mplus. Struct. Equ. Model..

[51] Nylund K.L., Asparouhov T., Muthén B.O. (2007). Deciding on the number of classes in latent class analysis and growth mixture modeling: A Monte Carlo simulation study. Struct. Equ. Model..

[52] Berlin K.S., Williams N.A., Parra G.R. (2014). An introduction to latent variable mixture modeling (part 1): Overview and cross-sectional latent class and latent profile analyses. J. Pediatr. Psychol..

[53] Nagin D.S. (2005). Group Based Modeling of Development.

[54] Asparouhov T., Muthén B. (2015). Residual associations in latent class and latent transition analysis. Struct. Equ. Model..

[55] Weigt J.M., Solomon C.R. (2008). Work–family management among low-wage service workers and assistant professors in the USA: A comparative intersectional analysis. Gend. Work Organ..

[56] Dickson M., Harmon C. (2011). Economic returns to education: What we know, what we don’t know, and where we are going—Some brief pointers. Econ. Educ. Rev..

[57] Kim K., Kye S. (2018). A study on work–family balance and the happiness level of dual career families. J. Korean Home Manag. Assoc..

[58] Solomon B.C., Nikolaev B.N., Shepherd D.A. (2022). Does educational attainment promote job satisfaction? The bittersweet trade-offs between job resources, demands, and stress. J. Appl. Psychol..

[59] Li J.-B., Dou K. (2021). Low involvement and ineffective monitoring link mothers’ work–family conflict and adolescent self-control. J. Fam. Issues.

[60] Zhang J. (2016). Work–Family Conflict and Child Well-Being: When Work–Family Conflict Hits Home. Doctoral Dissertation.

[61] Huyghebaert-Zouaghi T., Morin A.J.S., Fernet C., Austin S., Gillet N. (2022). Longitudinal profiles of work–family interface: Their individual and organizational predictors, personal and work outcomes, and implications for onsite and remote workers. J. Vocat. Behav..

